# EQTL analyses are a formidable tool to define the immunogenetic mechanisms underpinning Spondyloarthropathies

**DOI:** 10.3389/fimmu.2025.1518658

**Published:** 2025-03-18

**Authors:** Matteo Vecellio, Carlo Selmi

**Affiliations:** ^1^ Centro Ricerche Fondazione Italiana Ricerca in Reumatologia (FIRA), Fondazione Pisana per la Scienza ONLUS, San Giuliano Terme, Italy; ^2^ Wellcome Centre for Human Genetics, University of Oxford, Oxford, United Kingdom; ^3^ Department of Rheumatology and Clinical Immunology, IRCCS Humanitas Research Hospital, Milan, Italy; ^4^ Department of Biomedical Sciences, Humanitas University, Milan, Italy

**Keywords:** spondylitis, transcriptomics, genomics, spondyloarthropathies, genetics

In the practice of clinical rheumatology, identifying individuals at higher risk to develop a chronic inflammatory disease based on their genetic profiles remains a major wish. In the case of axial spondyloarthritis (axSpA) (1), the strong association with HLA-B27 may well fulfil this expectation but the vast prevalence of this allele in the general population makes in unsuitable for the early diagnosis. With better genetic tools available, clinicians may tailor more effective therapeutic interventions, based on the molecular pathways involved.

## Understanding eQTLs

EQTLs are genomic loci that modulate the expression of genes. Notably, these genetic factors are prevalently non-coding variants that can act across cell types and states (2). The most common method of analyzing such impact is to directly assay the levels of RNA produced from the gene of interest in connection with a specific genetic variant, done on a single transcript level via quantitative real-time PCR (qRT-PCR), or on a transcriptome-wide manner with RNA sequencing (RNA-seq) based methodologies (3). Therefore, eQTLs are genetic variants with validated functional effects on the differential expression of genes, and are linked to disease in ways similar to GWAS variants. There are crucial steps that must be followed to process eQTL data, including quality control, mean aggregation, covariate correlation procedures, and multiple testing corrections (4).

It is critical for this sort of approach to map eQTLs in the context of disease to link the direct contribution of specific regulatory variants to the disease pathogenesis (5). EQTLs can be classified into two main types: cis-eQTLs, which affect genes located nearby on the same chromosome, and trans-eQTLs, influencing genes situated far away on the genome or on different chromosomes. It’s not trivial to identify eQTLs indicative of genetic variations really contributing to disease mechanisms.

For eQTL mapping to provide disease insights, changes in RNA expression levels must be assayed in the specific cell types and conditions relevant to the disease of interest (6). The context specificity of eQTL effects it’s a pivotal concept because the transcriptome and its regulatory mechanisms are dynamic and very frequently context-dependent (7). Further, seminal studies have demonstrated that eQTLs may only be detected in certain cell types or upon stimulation, introducing the concept of response eQTLs (8), which connect disease-relevant treatments to genetic variation. Response eQTLs have the power to show how common genetic variation might contribute to gene expression changes only in activated conditions, such as a specific stimulus as shown in osteoarthritis (with a cartilage matrix breakdown product, fibronectin fragment), allowing the investigation of early stages of the disease (9, 10) It has been also demonstrated that GWAS associations have only limited overlap with eQTLs (11). The regulatory activity of the variants may be likely active only in disease relevant conditions, such as following immune activation, as it occurs in axSpA.

## eQTLs and Spondyloarthropathies

Several works have proven that eQTLs can significantly impact the expression of genes associated with inflammatory processes (12, 13). In axSpA, previously identified as ankylosing spondylitis, specific eQTLs have been linked to genes involved in the immune response and bone remodeling (14, 15). Back in 2016, Ellinghaus and colleague published a cross-disease study where they simultaneously investigated the genetic landscape and the pleiotropy of five clinically related conditions including axSpA, psoriasis, Crohn’s disease, primary sclerosing cholangitis, and ulcerative colitis. The authors were able to identify new coding variants and eQTLs for Fc Gamma Receptor IIα (*FCGR2A*), Endoplasmic reticulum aminopeptidase 2 (*ERAP2*), tyrosine-protein kinase 2 (*TYK2*) and fucosyltransferase 2 (*FUT2*) shared by the different diseases (16). Very importantly, variations in the *IL23R* gene, which encodes a receptor for interleukin 23 (*IL23R*) have been identified as potential eQTLs that may influence susceptibility to axSpA, although no convincing effects arising from the primary associated SNPS *rs11209032* have been observed on the transcription of either IL23R (and cognate IL12RB2) (17). On this matter, eQTL libraries generation as described by Fairfax et al. for monocytes might be very helpful, but they are highly cell specific and may therefore not be relevant to the disease context (8). More recently, Roberts and colleagues found that an intergenic SNP in the *IL23R*-*IL12RB2* region had a regulatory effect but despite no apparent regulation on the mRNA gene expression, the proportion of IFN-γ+ CD4+ T-cells was increased in the homozygote subjects carrying the disease genotype ‘A/A’ (18). It is very likely that other regulatory mechanisms are acting on this locus but the identification of eQTLs relevant to axSpA may help the researchers in shedding the light on the pathophysiology of the disease, also highlighting potential therapeutic targets.

From this perspective, Brown and colleagues reported different eQTLs, in the comprehensive genomic analysis performed on immune cells obtained from the peripheral blood of patients with axSpA (19). A total of nine loci, including Runt-related transcription factor 3 (*RUNX3*), Interleukin 7 receptor (*IL7R*, encoding the IL-7Rα subunit), ETS proto-oncogene 1 and 2 (*ETS1*, *ETS2*) and *B3GNT2* [previously associated with increased AS risk (14)], showed chromatin interaction, differential open chromatin profile, peaks for specific regulatory histone marks (such as H3K4me3 and H3K27ac) enhancer RNA (eRNA) peaks, and five of them overlapped eQTLs in the same cell types. Further, the authors demonstrated allele-specific differences in the ATAC-seq signal (Assay for Transposase-Accessible Chromatin using sequencing to identify open chromatin regions) (20) for an axSpA GWAS-associated SNP, *rs4672505*, previously associated with differential abundance of *B3GNT2*, a poly-N-acetyllactosamine synthase enzyme important in modulating T cell activation in cancer (21). A reduction of B3GNT2 expression in individuals with the axSpA risk allele is caused by a reduced chromatin openness at this locus. The authors suggest that this strong effect might impact other higher-order chromatin structures, such as the looping event identified they identified between a distal enhancer and B3GNT2 (19).

Back in 2018, the group of Professor Gaffney identified shared QTLs for chromatin accessibility and gene expression in human macrophages exposed to specific stimuli such as IFNγ, *Salmonella* and IFNγ + *Salmonella*. Understanding how genetic variants affect the binding of specific transcription factors, altering chromatin accessibility and enhancers behaviors during immune activation is central in untangling the molecular architecture of disease-associated variants (22) and so, to develop strategies to mediate the inflammatory processes (such as in axSpA).

## Single-cell-resolution eQTL as a new way in SpA modelling

Single cell (sc) technology has been revolutionary in the field of molecular biology and genomics, measuring the abundance of mRNA molecules per cell, detecting transcriptomic profiles at unprecedented scale and resolution (23, 24). Thanks to the development of sc-eQTL models that can identify cell-state-specific genetic effects on gene expression, it is now possible to define how genetic variants influence gene expression at the single-cell level and how genetic regulation varies in a dynamic way along specific trajectories. This approach is already very powerful in integrating existing knowledge about disease alleles with their predicted regulatory targets in defined cellular context. Sc profiling will be critical for studies of QTLs affecting the magnitude of dynamic expression responses together with their rate and timing (7). A recent study demonstrated the power of sc-eQTL mapping in linking genetic variants to complex diseases (i.e. neurological disorders): the authors performed sc-eQTL mapping on 196 individuals in eight central nervous system cell types and identified thousands of genes having cell-type-specific effects (25).

Transcriptomic analysis at single cell resolution is just the first layer of the complex regulatory machinery that controls functions and signaling in the cell. Other readouts, including chromatin accessibility, surface proteins, T cell receptor (TCR)/B cell receptor (BCR) repertoires, are measured to complement transcriptomic analysis at sc resolution. Simone and colleagues have recently identified multiple Tregs clusters in the peripheral blood and synovial fluid of patients with axSpA. Of note, one regulatory CD8+ subset expressed cytotoxic markers, and a Th17-like RORC+ Treg subset was specifically characterized by the expression of the immunomodulatory molecule lymphocyte activating 3 (LAG-3) (26). The value of using patient blood and/or tissue derived cells such as synovial tissue/fluid to perform scRNA-seq-based eQTL studies is undeniable (6). We must think differently: instead of treating individuals as observations, trajectories models treat each cell as its own observation of the expression of a gene. Few years ago, Perez and colleagues published a seminal work where they profiled more than 1 million of circulating immune cells with multiplexed sc-RNA-seq. Integrating these data with genotyping data the authors map cell type– and cell context–specific cis-eQTLs that might mediate systemic lupus erythematosus (SLE) disease associations and have a plausible functional effect. Joint analysis of cis-eQTLs and genome-wide association study results enabled the identification of cell types relevant to immune-mediated diseases, fine-mapping of disease-associated loci, and discovery of novel SLE associations (27). Furthermore, Yazar and colleagues showed through a population-based study how segregating alleles may contribute to variation in the function of 14 immune cell types. With the integration of these data with autoimmune disease (including RA, SLE and AxSpA) cohorts the authors identified causal effects for more than 160 loci (28). Of note, although sc-transcriptomics offers a vast opportunity to expand eQTL discovery and map in depth transcriptional regulation, several methodological limitations still remain. Sc-technology is not exempt from batch effects, and data might still be influenced by technical factors. Nevertheless, different computational batch correction methods are available, with the aim to remove technical variation from the data, thus preventing the variation from confounding downstream analysis (29). Of note, there are rare or particular cell types (i.e. neutrophils with high levels of RNases) which are still challenging to measure, accentuating a need in improving annotation efficiency, cell state definitions and correlation analysis. Further, standard pipelines lack the power to accurately quantify specific genes (i.e. HLA genes, which are very polymorphic across individuals): this suggests there are instances for the development of more specialized gene quantification pipelines (30).

## Implications for research and treatment

Studying eQTLs might open new opportunities for personalized medicine in axSpA. Understanding the molecular pathways influenced by eQTLs can facilitate the development of novel therapeutics aimed at modulating gene expression or targeting specific inflammatory pathways. As previously indicated, Brown and colleagues combined their genomics analysis (19) with PI (priority index, a genetics-led approach that interprets GWASs with functional genomic data to prioritize therapeutic targets in immune-mediated and inflammatory disorders) (31). The authors successfully confirmed pathways known to be important in AS pathogenesis, such as Th17/IL-23 and TNF, and identified novel pathways and potential drug targets, such as PTGER4, phosphatidylinositol 3-kinase (PI3K)/AKT, NOTCH, ErbB and GPCR. The link between a particular eQTL variant and the increased expression of a disease-associated gene, suggests that modulating its expression might have therapeutic benefits. Existing inhibitors of the pathways identified in the AS comprehensive study, such as the PI3K inhibitors currently used for the treatment of lymphoma, could be repurposed, and optimized in axSpA. Further, the identification of multiple eQTLs can contribute to develop combination therapies by identifying those genes that work synergistically in disease pathways. On this line, Goldmann et al. performed eQTL analysis in synovial tissue and blood samples obtained from treatment-naïve patients with rheumatoid arthritis (RA). In particular the authors identified 898 eQTL in synovium, and 1251 eQTL in peripheral blood, Among these, a specific eQTL at HLA-DPB2 gene was discovered, with the genetic variant *rs3128921* driving synovial HLA-DPB2 expression. Both *rs3128921* and HLA-DPB2 expression correlated with disease severity and lympho-myeloid pathotype, indicating a potential for immediate aggressive treatment stratification (10).

The multimodal approaches we have briefly discussed here, consisting in assessing gene expression, open chromatin identification and chromatin interactions, can inform the interpretation of axSpA GWAS indicating the likely functional variants and the gene regulatory networks to prioritize for the identification of new potential drug targets.

## Concluding remarks

EQTLs represent a promising frontier in the genetic research of axSpA. By elucidating the connections between genetic variation, gene expression, and disease mechanisms, eQTL studies hold the potential to enhance our understanding of SpA and improve patient outcomes through more personalized treatment approaches. Future research should focus on integrating eQTL data with other omics approaches, such as proteomics and metabolomics, to create a more comprehensive understanding of gene regulation. Recently, Zhao and colleagues performed a comprehensive proteome-wide mendelian randomization study to assess the causal relationships between plasma proteins and increased SpA susceptibility, to identify potential therapeutic targets and to repurpose licensed drugs (32). The utility of metabolomic profiling integrated with transcriptomic and genomics is indisputed, as it might help to understand molecular mechanisms for metabolite levels in diseases (33). The so-called metabQTLs, the genetic associations discovered through GWAS with metabolite levels, add another layer of complexity to the full picture but are of extreme value to gain metabolic insights and facilitate the discovery of disease-associated molecular mechanisms (34). Metabolomics and proteomics analyses are widely applied for new biomarkers discovery: both approaches must be considered for the comprehensive identification of the metabolic changes occurring in SpA.

As previously anticipated, advancements in sc-RNA-seq technology also hold the potential to dissect eQTL effects at the cellular level, revealing insights into cell-type-specific gene expression patterns and allowing patients with the same clinical disease to be stratified into subgroups for better therapeutic strategies (35).

However, the identification of eQTLs is just the first step, as understanding their functional consequences is the real critical point. Validating the biological impact of identified eQTLs through functional assays, such as CRISPR/Cas9 gene editing or RNA interference is crucial to better clarify how specific genetic variants influence gene expression (36) and contribute to disease, providing a more robust basis for therapeutic development (37). The evaluation of the functional role of each specific SNP or haplotype (a set of genetic determinants inherited together) (38) is fundamental to provide biological evidence to support the susceptibility of a genomic locus. So, perturbing the expression of genes (gene overexpression and gene silencing) with a CRISPR based approach (CRISPR KO, CRISPR activation and CRISPR interference) has the power to assess the regulatory effect of a specific genetic variant (39). Further, chromosome conformation capture techniques (Hi-C, Micro-C and Micro Capture C) are also able to resolve (40) GWAS signals to functionally correlate disease-associated SNPs with target genes and thus explain differential gene expression (41). Assessing SNP regulatory functions include also the investigation of the effects on protein-DNA complex formation, as the regulatory effects of transcription factors are mediated through complexes binding to DNA regulatory regions, associated to euchromatin (open active chromatin) (39, 42).

Artificial intelligence (AI) and machine learning techniques can be also applied to significantly enhance eQTL research, as these technologies can analyze large datasets more efficiently, uncovering complex patterns and interactions that might be missed by traditional analytical methods (43). AI can also facilitate the development of predictive models that integrate genetics, epigenomics, and phenotypic data to anticipate disease risk and treatment outcomes, together with the identification of novel drug targets and the optimization of existing therapies.
